# Supplementary material for: Anti-viral defence by an mRNA ADP-ribosyltransferase that blocks translation
Source: Nature. 2024 Oct 23;636(8041):190–7. doi: 10.1038/s41586-024-08102-8 (PMC11618068; doi:10.1038/s41586-024-08102-8)
Supplement: Supplementary file 1 — Supplementary Fig. 1 and Supplementary Tables 1–4. [file 41586_2024_8102_MOESM1_ESM.pdf]

nature portfolio

https://doi.org/10.1038/s41586-024-08102-8

Supplementary information

Anti-viral defence by an mRNA ADP-ribosyltransferase that blocks translation

In the format provided by the authors and unedited

SI Guide

Supplementary Fig. 1: Uncropped blot images shown in Main and Extended Data figures. Uncropped images are labeled with their corresponding Main or Extended Data figure number as well as the membrane or gel type and the staining method or antibody used in detection. Blots shown are Western blots on PVDF membranes unless otherwise stated. Note that for immunoblots, membranes were first stained and imaged to determine molecular weight markers and then probed and imaged separately by chemiluminescence. Images were aligned to relate chemiluminescent bands to molecular weight markers, as shown in main figures.

Supplementary Table 1: High confidence IP-MS/MS with spectral counts for CmdC and CmdT pulldown experiments with untagged control.

Supplementary Table 2: List of all oligonucleotides and primers used in this study.

Supplementary Table 3: List of all strains used in this study.

Supplementary Table 4: List of all plasmids used in this study.

Fig. 2b

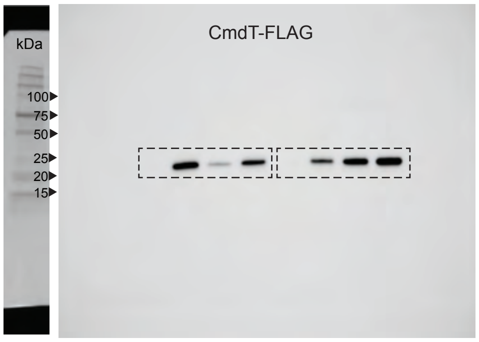

Fig. 2b

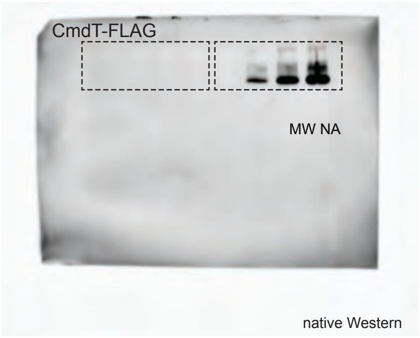

Fig. 2c

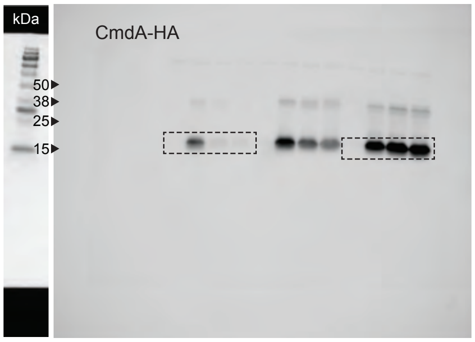

Fig. 2d (and ED Fig. 4e)

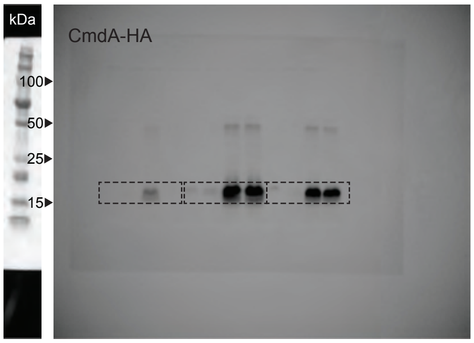

Fig. 2e

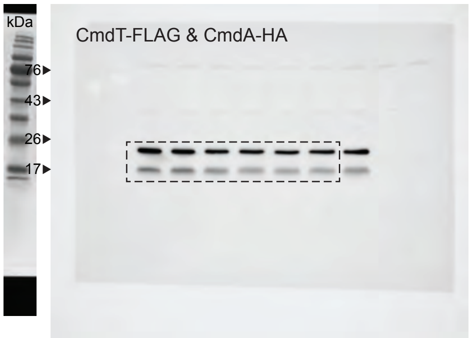

Fig. 2g

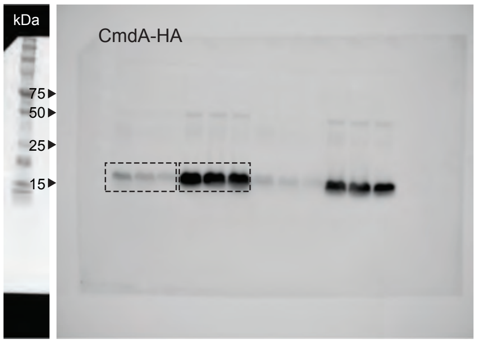

Fig. 3a

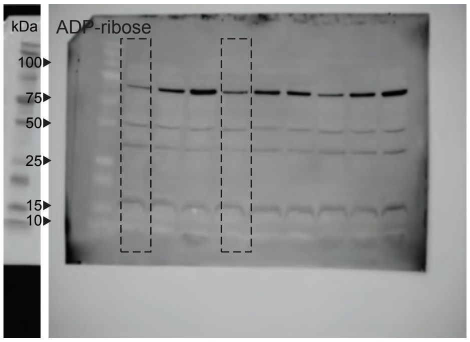

Fig. 3a

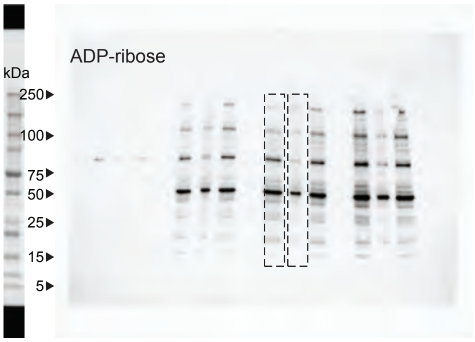

Fig. 3a

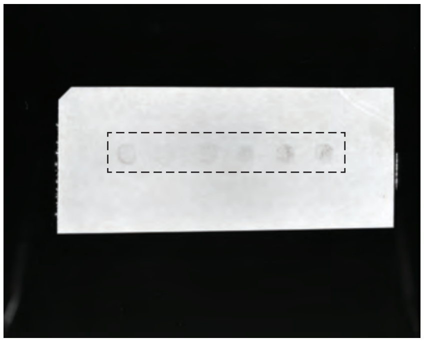

Fig. 3a

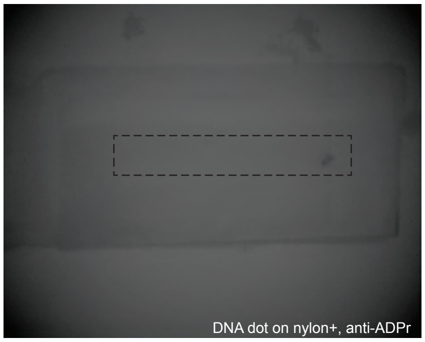

Fig. 3a

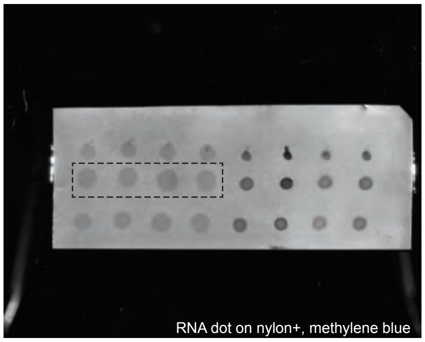

Fig. 3a

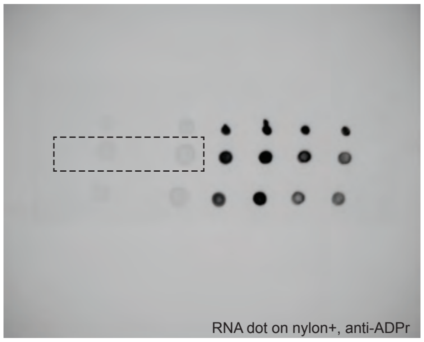

Fig. 3c

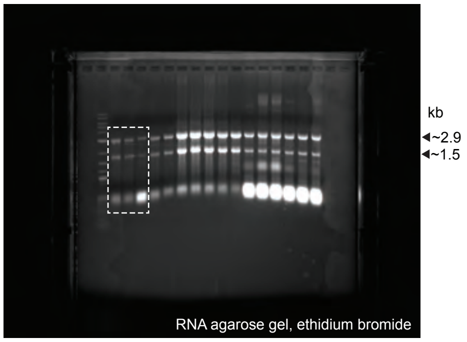

Fig. 3c

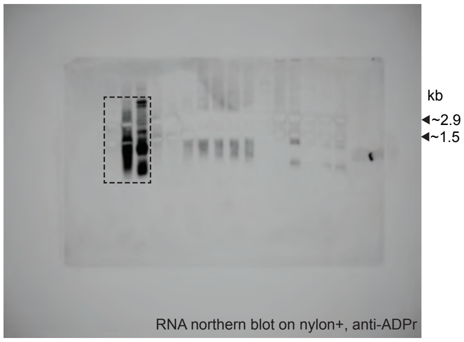

Fig. 3d

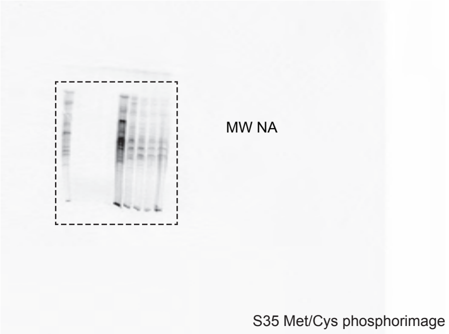

Fig. 3e

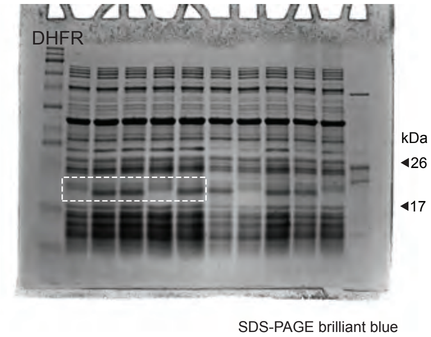

Fig. 3f

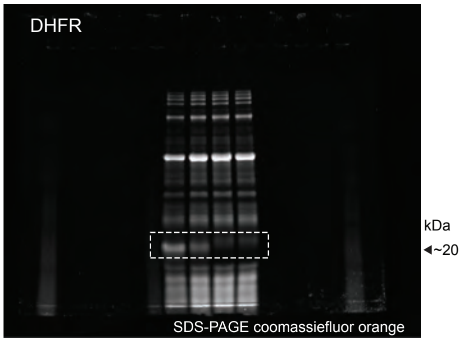

Fig. 4a

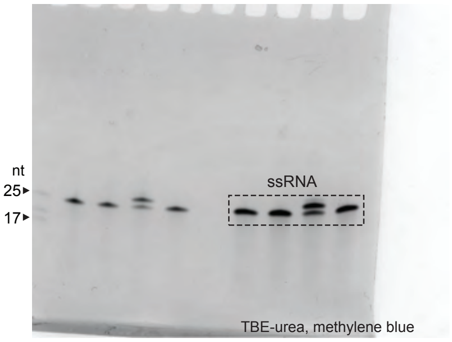

Fig. 4b

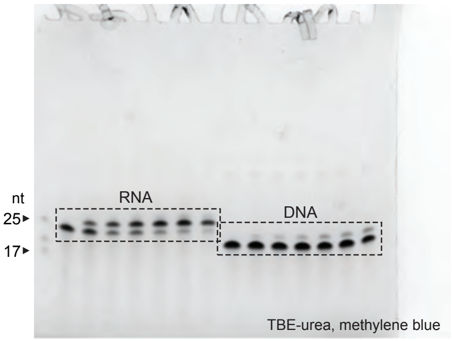

Fig. 4c

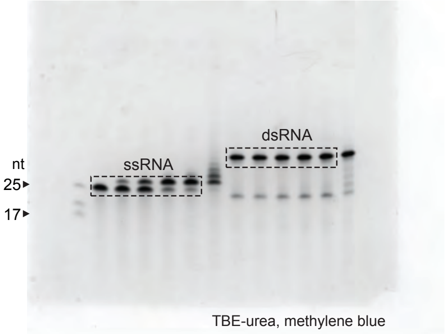

Fig. 4d

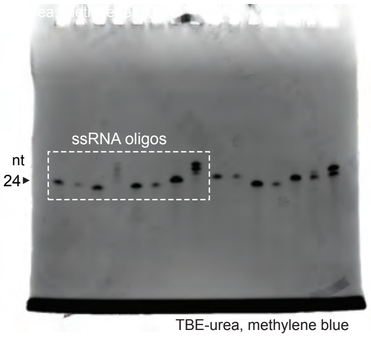

Fig. 4e

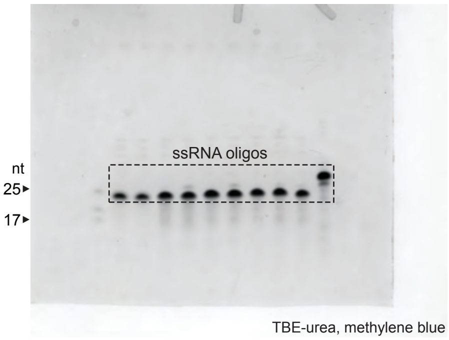

Extended Data Fig. 4e

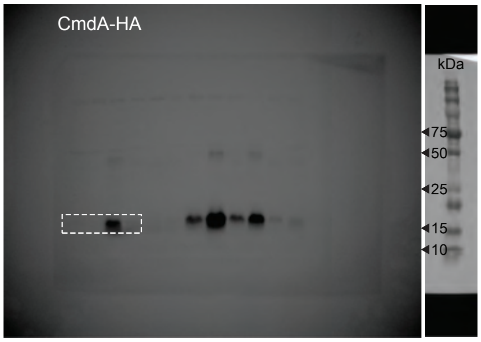

Extended Data Fig. 3d

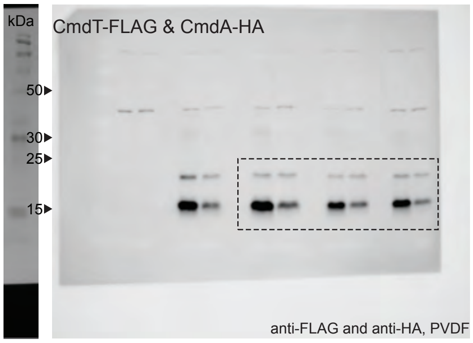

Extended Data Fig. 3d

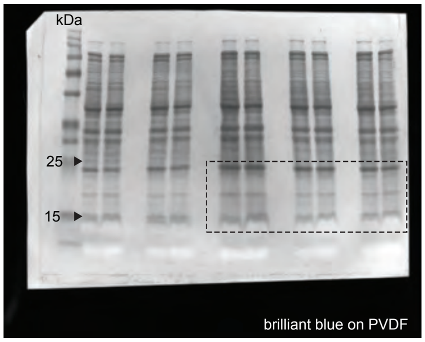

Extended Data Fig. 5a

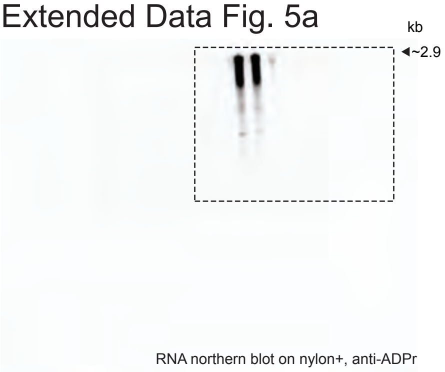

Extended Data Fig. 5a

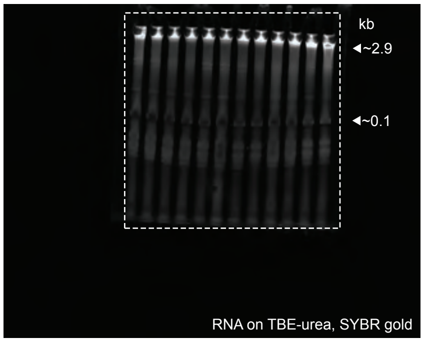

Extended Data Fig. 5b

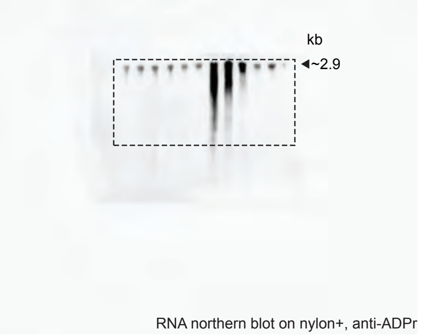

Extended Data Fig. 5b

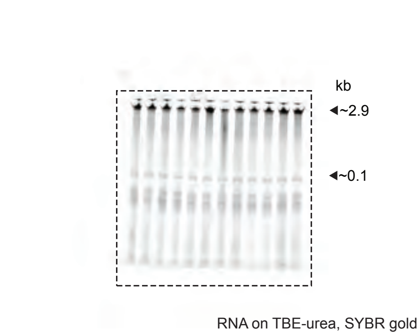

Extended Data Fig. 5c

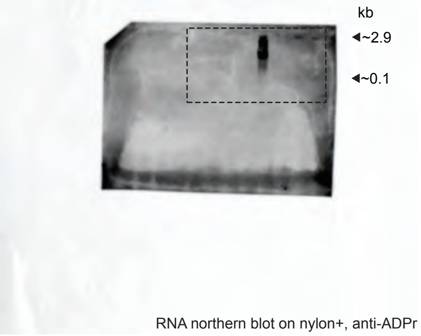

Extended Data Fig. 5c

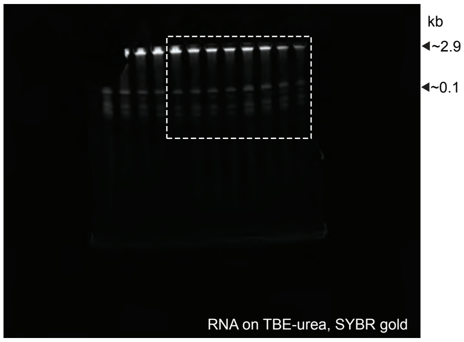

Extended Data Fig. 6a

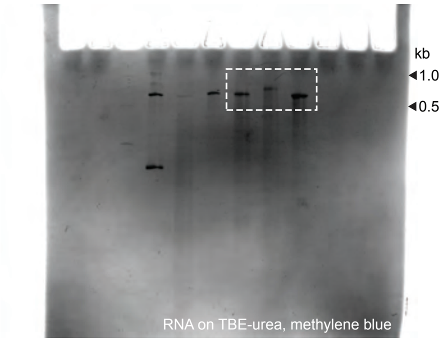

Extended Data Fig. 6a

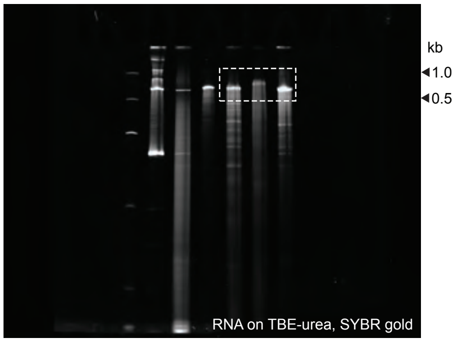

Extended Data Fig. 6d

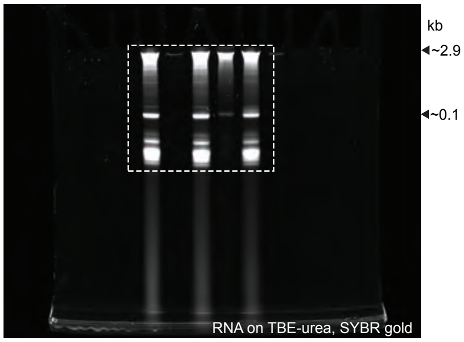

Extended Data Fig. 7a

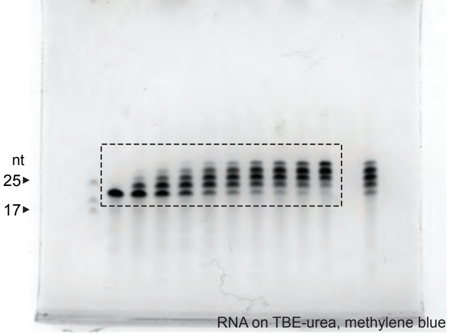

SI Table 1: CmdT and CmdC IP-MS/MS High Confidence Hits

		# PSMs (by Search Engine)					
**Accession**	**Description**	**CmdC IP 0 min**	**CmdC IP 15 min**	**Control 0 min**	**Control 15 min**	**CmdT IP 0 min**	**CmdT IP 15 min**
P0CE48	Elongation factor Tu 2 OS=Escherichia coli (strain K12) OX=83333 GN=tufB PE=1 SV=1	83	94	68	68	85	73
P31120	Phosphoglucosamine mutase OS=Escherichia coli (strain K12) OX=83333 GN=glmM PE=1 SV=3	63	61	58	59	49	56
P0A7V3	30S ribosomal protein S3 OS=Escherichia coli (strain K12) OX=83333 GN=rpsC PE=1 SV=2	37	40	53	55	72	45
P0ADZ0	50S ribosomal protein L23 OS=Escherichia coli (strain K12) OX=83333 GN=rplW PE=1 SV=1	26	21	19	64	97	47
P61175	50S ribosomal protein L22 OS=Escherichia coli (strain K12) OX=83333 GN=rplV PE=1 SV=1	26	29	32	49	39	39
P0A7S3	30S ribosomal protein S12 OS=Escherichia coli (strain K12) OX=83333 GN=rpsL PE=1 SV=2	24	24	35	39	38	41
P0A707	Translation initiation factor IF-3 OS=Escherichia coli	27	24	23	45	37	32

	(strain K12) OX=83333 GN=infC PE=1 SV=1						
P60624	50S ribosomal protein L24 OS=Escherichia coli (strain K12) OX=83333 GN=rplX PE=1 SV=2	20	18	19	42	50	34
P60723	50S ribosomal protein L4 OS=Escherichia coli (strain K12) OX=83333 GN=rplD PE=1 SV=1	19	17	19	40	42	26
P62399	50S ribosomal protein L5 OS=Escherichia coli (strain K12) OX=83333 GN=rplE PE=1 SV=2	25	22	30	31	32	19
A0A7S9SVH2	Internal head protein OS=Escherichia phage T4 OX=2681598 GN=ipIII PE=4 SV=1		43		40		40
CDCMDCC	CmdC Chaperone	33	33			43	38
P0A7U7	30S ribosomal protein S20 OS=Escherichia coli (strain K12) OX=83333 GN=rpsT PE=1 SV=2	15	16	27	33	31	26
P0A7M2	50S ribosomal protein L28 OS=Escherichia coli (strain K12) OX=83333 GN=rpmB PE=1 SV=2	17	19	25	34	31	27
P0A7V8	30S ribosomal protein S4 OS=Escherichia coli (strain K12) OX=83333 GN=rpsD PE=1 SV=2	19	19	19	30	31	26
P0A9X4	Cell shape-determining protein MreB OS=Escherichia coli (strain	31	26	22	26	19	17

	K12) OX=83333 GN=mreB PE=1 SV=1						
P0A7W1	30S ribosomal protein S5 OS=Escherichia coli (strain K12) OX=83333 GN=rpsE PE=1 SV=2	13	26	23	26	37	25
P0A7U3	30S ribosomal protein S19 OS=Escherichia coli (strain K12) OX=83333 GN=rpsS PE=1 SV=2	18	18	25	20	36	17
P68679	30S ribosomal protein S21 OS=Escherichia coli (strain K12) OX=83333 GN=rpsU PE=1 SV=2	20	17	20	26	23	20
CDCMDT	CmdT Toxin	12	3			58	63
P04264	Keratin, type II cytoskeletal 1 OS=Homo sapiens OX=9606 GN=KRT1	13	9	10	5	29	86
P60422	50S ribosomal protein L2 OS=Escherichia coli (strain K12) OX=83333 GN=rplB PE=1 SV=2	16	25	13	25	8	18
P0AG55	50S ribosomal protein L6 OS=Escherichia coli (strain K12) OX=83333 GN=rplF PE=1 SV=2	8	9	12	28	32	28
P02413	50S ribosomal protein L15 OS=Escherichia coli (strain K12) OX=83333 GN=rplO PE=1 SV=1	15	14	15	23	23	20
P0CG19	Truncated inactive ribonuclease PH OS=Escherichia coli (strain	26	16	22	17	19	13

	K12) OX=83333 GN=rph PE=1 SV=1						
P0A7L8	50S ribosomal protein L27 OS=Escherichia coli (strain K12) OX=83333 GN=rpmA PE=1 SV=2	16	11	14	21	24	21
P02359	30S ribosomal protein S7 OS=Escherichia coli (strain K12) OX=83333 GN=rpsG PE=1 SV=3	18	14	10	14	13	23
P00761	Trypsin OS=Sus scrofa OX=9823	19	17	15	16	17	16
P0A7R9	30S ribosomal protein S11 OS=Escherichia coli (strain K12) OX=83333 GN=rpsK PE=1 SV=2	15	15	13	18	19	15
P0A7V0	30S ribosomal protein S2 OS=Escherichia coli (strain K12) OX=83333 GN=rpsB PE=1 SV=2	11	9	13	18	25	17
P0A9M8	Phosphate acetyltransferase OS=Escherichia coli (strain K12) OX=83333 GN=pta PE=1 SV=2	15	13	18	13	22	14
P0AA10	50S ribosomal protein L13 OS=Escherichia coli (strain K12) OX=83333 GN=rplM PE=1 SV=1	13	12	11	17	31	13
P0C018	50S ribosomal protein L18 OS=Escherichia coli (strain K12) OX=83333 GN=rplR PE=1 SV=1	11	13	11	19	21	16
P0AG59	30S ribosomal protein S14 OS=Escherichia coli (strain	11	12	11	16	15	15

	K12) OX=83333 GN=rpsN PE=1 SV=2						
P0AG44	50S ribosomal protein L17 OS=Escherichia coli (strain K12) OX=83333 GN=rplQ PE=1 SV=1	5	15	20	12	26	11
P49065	Albumin OS=Oryctolagus cuniculus OX=9986 GN=ALB	12	15	7	12	21	14
P0A8F4	Uridine kinase OS=Escherichia coli (strain K12) OX=83333 GN=udk PE=3 SV=1	9	15	3	16		17
P0A7T3	30S ribosomal protein S16 OS=Escherichia coli (strain K12) OX=83333 GN=rpsP PE=1 SV=1	9	9	10	15	16	14
P0ADY3	50S ribosomal protein L14 OS=Escherichia coli (strain K12) OX=83333 GN=rplN PE=1 SV=1	6	7	11	20	15	15
P02358	30S ribosomal protein S6 OS=Escherichia coli (strain K12) OX=83333 GN=rpsF PE=1 SV=1	10	11	9	12	15	11
P0AG48	50S ribosomal protein L21 OS=Escherichia coli (strain K12) OX=83333 GN=rplU PE=1 SV=1	6	37	8	11	9	6
P0ADY7	50S ribosomal protein L16 OS=Escherichia coli (strain K12) OX=83333 GN=rplP PE=1 SV=1	9	10	12	12	12	12

P0A7K6	50S ribosomal protein L19 OS=Escherichia coli (strain K12) OX=83333 GN=rplS PE=1 SV=2	6	7	9	20	18	7
CDCMDA	CmdA Antitoxin	7	3			30	30
A0A7S9SVU2	Internal head protein OS=Escherichia phage T4 OX=2681598 GN=ipII PE=4 SV=1		9		18		24
P08622	Chaperone protein DnaJ OS=Escherichia coli (strain K12) OX=83333 GN=dnaJ PE=1 SV=3		18		18		17
P0A7X3	30S ribosomal protein S9 OS=Escherichia coli (strain K12) OX=83333 GN=rpsI PE=1 SV=2	8	7	10	10	15	9
P0A7N9	50S ribosomal protein L33 OS=Escherichia coli (strain K12) OX=83333 GN=rpmG PE=1 SV=2	9	7	6	11	9	11
P35908	Keratin, type II cytoskeletal 2 epidermal OS=Homo sapiens OX=9606 GN=KRT2	3	2	3	1	38	26
P0A7L0	50S ribosomal protein L1 OS=Escherichia coli (strain K12) OX=83333 GN=rplA PE=1 SV=2	9	10	4	10	2	7
P35527	Keratin, type I cytoskeletal 9 OS=Homo sapiens OX=9606 GN=KRT9	3	4	1		3	56
P0A7R5	30S ribosomal protein S10 OS=Escherichia coli (strain	8	5	7	8	11	5

	K12) OX=83333 GN=rpsJ PE=1 SV=1						
P13645	Keratin, type I cytoskeletal 10 OS=Homo sapiens OX=9606 GN=KRT10	4	2	2	2	29	25
P60438	50S ribosomal protein L3 OS=Escherichia coli (strain K12) OX=83333 GN=rplC PE=1 SV=1	4	3	5	15	13	8
P00960	Glycine--tRNA ligase alpha subunit OS=Escherichia coli (strain K12) OX=83333 GN=glyQ PE=1 SV=2	6	9	8	6	6	6
P0A910	Outer membrane protein A OS=Escherichia coli (strain K12) OX=83333 GN=ompA PE=1 SV=1	11	1	6		11	7
P0AG67	30S ribosomal protein S1 OS=Escherichia coli (strain K12) OX=83333 GN=rpsA PE=1 SV=1	1	1				20
P0AAH0	Phosphate import ATP-binding protein PstB OS=Escherichia coli (strain K12) OX=83333 GN=pstB PE=1 SV=2	9	6	6	10	3	5
A0A7S9SW12	Single-stranded DNA-binding protein OS=Escherichia phage T4 OX=2681598 PE=4 SV=1		10		11		9
P0A7T7	30S ribosomal protein S18 OS=Escherichia coli (strain K12) OX=83333 GN=rpsR PE=1 SV=2	4	4	6	5	9	7

P0ADZ4	30S ribosomal protein S15 OS=Escherichia coli (strain K12) OX=83333 GN=rpsO PE=1 SV=2	5	5	4	8	7	5
P08779	Keratin, type I cytoskeletal 16 OS=Homo sapiens OX=9606 GN=KRT16	4	2	2	2	8	24
P0A7J7	50S ribosomal protein L11 OS=Escherichia coli (strain K12) OX=83333 GN=rplK PE=1 SV=2	4	1	4	4	7	4
P02533	Keratin, type I cytoskeletal 14 OS=Homo sapiens OX=9606 GN=KRT14	4	2	2	2	8	20
A0A7S9SVJ4	Major capsid protein OS=Escherichia phage T4 OX=2681598 PE=3 SV=1		10				1
A0A7S9XGF4	Uncharacterized protein OS=Escherichia phage T4 OX=2681598 PE=4 SV=1		6		5		9
P13647	Keratin, type II cytoskeletal 5 OS=Homo sapiens OX=9606 GN=KRT5		1			7	30
A0A7S9SVS8	Alpha glucosyl transferase OS=Escherichia phage T4 OX=2681598 GN=a-gt PE=4 SV=1		6		5		4
A0A7S9SVX7	Endolysin OS=Escherichia phage T4 OX=2681598 GN=e PE=3 SV=1	2	3	2	4	7	4
P0A7J3	50S ribosomal protein L10 OS=Escherichia coli (strain	5	6	1	4	2	2

	K12) OX=83333 GN=rplJ PE=1 SV=2						
P60785	Elongation factor 4 OS=Escherichia coli (strain K12) OX=83333 GN=lepA PE=1 SV=1	4	3	2	4	5	4
P0A717	Ribose-phosphate pyrophosphokinase OS=Escherichia coli (strain K12) OX=83333 GN=prs PE=1 SV=2	4	1	4	4	5	3
A0A7S9SVR7	DNA topoisomerase (ATP-hydrolyzing) OS=Escherichia phage T4 OX=2681598 PE=4 SV=1		5		10		6
P21829	Pyridoxal phosphate phosphatase YbhA OS=Escherichia coli (strain K12) OX=83333 GN=ybhA PE=1 SV=3	3	2	5	3	3	3
P02538	Keratin, type II cytoskeletal 6A OS=Homo sapiens OX=9606 GN=KRT6A	1		1		6	18
P0A7S9	30S ribosomal protein S13 OS=Escherichia coli (strain K12) OX=83333 GN=rpsM PE=1 SV=2		1	3	1	10	2
A0A7S9XGY1	RecA-like recombination protein OS=Escherichia phage T4 OX=2681598 GN=uvsX PE=3 SV=1		5		5		3
P0A7B8	ATP-dependent protease subunit HslV OS=Escherichia coli (strain		3		4		4

	K12) OX=83333 GN=hslV PE=1 SV=2						
P0A7G6	Protein RecA OS=Escherichia coli (strain K12) OX=83333 GN=recA PE=1 SV=2	3	1	2	1		2
P0AEU7	Chaperone protein Skp OS=Escherichia coli (strain K12) OX=83333 GN=skp PE=1 SV=1		2		5	4	3
A0A7S9SW92	Protector from prophage-induced early lysis OS=Escherichia phage T4 OX=2681598 GN=rIIB PE=4 SV=1		3		4		3
P62768	UPF0325 protein YaeH OS=Escherichia coli (strain K12) OX=83333 GN=yaeH PE=3 SV=1				6		4
P0A7M6	50S ribosomal protein L29 OS=Escherichia coli (strain K12) OX=83333 GN=rpmC PE=1 SV=1				3	3	4
P0AGD7	Signal recognition particle protein OS=Escherichia coli (strain K12) OX=83333 GN=ffh PE=1 SV=1				11	1	2
P0A7P5	50S ribosomal protein L34 OS=Escherichia coli (strain K12) OX=83333 GN=rpmH PE=1 SV=1				4	4	2
P19013	Keratin, type II cytoskeletal 4 OS=Homo	1		1		4	8

	sapiens OX=9606 GN=KRT4						
P0AG63	30S ribosomal protein S17 OS=Escherichia coli (strain K12) OX=83333 GN=rpsQ PE=1 SV=2				4	4	2
P0A8J4	UPF0250 protein YbeD OS=Escherichia coli (strain K12) OX=83333 GN=ybeD PE=1 SV=1		4				
A0A7S9XGF0	Uncharacterized protein OS=Escherichia phage T4 OX=2681598 PE=4 SV=1		10		1		
A0A7S9XI41	Uncharacterized protein OS=Escherichia phage T4 OX=2681598 PE=4 SV=1		3		3		2
P0AGL7	Ribosomal RNA small subunit methyltransferase E OS=Escherichia coli (strain K12) OX=83333 GN=rsmE PE=1 SV=1				3	2	3
P0A8Z0	Acyl-CoA thioester hydrolase YciA OS=Escherichia coli (strain K12) OX=83333 GN=yciA PE=3 SV=1	3		2		2	
P0A9P6	ATP-dependent RNA helicase DeaD OS=Escherichia coli (strain K12) OX=83333 GN=deaD PE=1 SV=2	1					7
P0A7L3	50S ribosomal protein L20 OS=Escherichia coli (strain K12) OX=83333 GN=rplT PE=1 SV=2				1		

P13646	Keratin, type I cytoskeletal 13 OS=Homo sapiens OX=9606 GN=KRT13					2	10
P0ABJ1	Cytochrome bo(3) ubiquinol oxidase subunit 2 OS=Escherichia coli (strain K12) OX=83333 GN=cyoA PE=1 SV=1	1		1		2	2
P69786	PTS system glucose-specific EIICB component OS=Escherichia coli (strain K12) OX=83333 GN=ptsG PE=1 SV=1	1	1	2			
P0C0R7	Ribosomal RNA large subunit methyltransferase E OS=Escherichia coli (strain K12) OX=83333 GN=rlmE PE=1 SV=1				1	7	
A0A7S9SU18	Uncharacterized protein OS=Escherichia phage T4 OX=2681598 PE=4 SV=1		2		2		1
A0A7S9SU76	Tail completion and sheath stabilizer protein OS=Escherichia phage T4 OX=2681598 PE=4 SV=1		3				
A0A7S9XEX1	Uncharacterized protein OS=Escherichia phage T4 OX=2681598 PE=4 SV=1						4
P19909	Immunoglobulin G-binding protein G OS=Streptococcus sp. group G OX=1320 GN=spg				1	1	

SI Table 2: Oligonucleotides and Primers

Oligo Number	Used For	Sequence (5'->3')
CD1	Linearize pKVS45, -P_{TET} for construction of pCD1-*cmdTAC*	ccgatggtagtgtggggtc
CD2	Linearize pKVS45, -P_{TET} for construction of pCD1-*cmdTAC*	agatcttttctcctctttgctagc
CD3	Cloning CmdTAC in pCD1	cgctagcaaagaggagaaaagat ctggaaactccttttattggtg
CD4	Cloning CmdTAC in pCD1	tggggagaccccacactaccatc ggtcaattgggaaactcacc
CD5	Linearize pBAD30	aagcttggctgttttggc
CD6	Linearize pBAD30	gagctcaatccctcctgaattc
CD7	Insert CmdT in pBAD30	agcgaattcaggagggattgagct cgtgcaggtagttcatatag
CD8	Insert CmdT in pBAD30	ctcatccgccaaaacagccaagct tttaaaccttctcacagaaatc
CD9	Add CmdA to CmdT in pBAD30	agaagatttctgtgagaaggtttaa ggagggaaaatgctttcctt
CD10	Add CmdA to CmdT in pBAD30	ctcatccgccaaaacagccaagct ttcaggccgctaaatccaatt
CD11	Insert CmdC in pIF	accttatcaaacgggactcaaatct atgaagctaagattaaggga
CD12	Insert CmdC in pIF	tggggagaccccacactaccatc ggtcaattgggaaactcaccga
CD13	Insert CmdC in pBAD30	agcgaattcaggagggattgagct catgaagctaagattaaggga

CD14	Insert CmdC in pBAD30	ctcatccgccaaaacagccaagcttcaattgggaaactcaccga
CD15	Insert CmdTA into pBAD30 with CD-7	ctcatccgccaaaacagccaagcttcaggccgctaaatccaatt
CD16	Isolate pUC19 origin to insert into pBAD30 to make pCD16	cgtataatatttgccgctagcggagttgctggcgtttttccataggctcc
CD17	Isolate pUC19 origin to insert into pBAD30 to make pCD16	ggctcgccacttcgggctcatgagcaaatcttgagatcctttttttctgcgcgtaatctg
CD18	Linearize pBAD30 without origin to make pCD16	atttgctcatgagcccgaagtgg
CD19	Linearize pBAD30 without origin to make pCD16	ctccgctagcggcaaatattatacgcaa
CD20	Insert cmdTAC into pBAD30	tacccgtttttttgggctagcgaattcagagaggatgattgtgcaggtagttcatatagg
CD21	Insert cmdTAC into pBAD30	cttctctcatccgccaaaacagccaagctttcaattgggaaactcaccgatatcttgttc
CD22	Insert CT FLAG tag to CmdT in pBAD30-*cmdTA*	atctttccagaagatttctgtgagaaggttgattataaagatgatgatgataaataaggagggaaaatgctttcctttgaacaa
CD23	Insert CT FLAG tag to CmdT in pBAD30-*cmdTA*	ttgttcaaaggaaagcattttccctccttatttatcatcatcatctttataatcaaccttctcacagaaatcttctggaaagat
CD24	Linearize pIF and pKVS45	ccgatggtagtgtggggtct

CD25	Linearize pIF and pKVS45	agatttgagtcccgtttgataaggtatggagaaacctaggtgctcagtatctc
CD26	Insert Alt.-3† into pIF	taccttatcaaacgggactcaaatctatgaaatcttctttacgctttttaggtcaagaac
CD27	Insert Alt.-3† into pIF	ggggagaccccacactaccatcggtcaattcgcagagtaaaatattaggagcaatataac
CD28	Insert Alt.-3 into pIF	cacctaggtttctccatacaggaggtacccatgaaatcttctttacgctttttaggtcaa
CD29	Insert Alt.-3 into pIF	tcgcatggggagaccccacactaccatcggttatttacggaatgaaatgaaagcagcaac
CD30	Insert ClpP into pBAD30	gggctagcgaattcaggagggattgagctcatgtcatacagcggcgaacgag
CD31	Insert ClpP into pBAD30	cttctctcatccgccaaaacagccaagctttcaattacgatgggtcagaatcgaatcgac
CD32	insert CmdA into pBAD30	gggctagcgaattcaggagggattgagctcatgctttcctttgaacaaagaatggcct
CD33	insert CmdA into pBAD30	cttctctcatccgccaaaacagccaagctttcaggccgctaaatccaattcagtatcatc
CD34	Insert T4 Gp23 into pCD16	gggctagcgaattcaggagggattgagctcatgactatcaaaactaaagctgaacttttgaacaaatgg

CD35	Insert T4 Gp23 into pCD16	cttctctcatccgccaaaacagcc aagcttttagatacctttaacatata cacgtctaaagtaagcg
CD36	insert T4 Gp31 into pKVS45	ctccataccttatcaaacgggactc aaatctatgtctgaagtacaacagc taccaattcg
CD37	insert T4 Gp31 into pKVS45	tcgcatgggggagaccccacacta ccatcggtcacttataaagacacg gaatagctttatagtgacagg
CD38	insert Alt.-3 or Alt.-3† into pBAD30	gggctagcgaattcaggagggat tgagctcatgaaatcttctttacgct ttttaggtcaa
CD39	insert Alt.-3 into pBAD30	cttctctcatccgccaaaacagcc aagcttttatttacggaatgaaatga aagcagcaac
CD40	insert Alt.-3† into pBAD30	cttctctcatccgccaaaacagcc aagctttcaattcgcagagtaaaat attaggagcaa
CD41	Insert CT FLAG tag to CmdT in pCD1-*cmdTAC* from pBAD30-*cmdT*$_{CT\text{-}FLAG}$*A*	gcttccaagctccgaagggttca
CD42	Insert CT FLAG tag to CmdT in pCD1-*cmdTAC* from pBAD30-*cmdT*$_{CT\text{-}FLAG}$*A*	tgaacccttcggagcttggaagc
CD43	Insert CT FLAG tag to CmdT in pCD1-*cmdTAC* from pBAD30-*cmdT*$_{CT\text{-}FLAG}$*A*	ggaacagatggtttcatttactcgt cagcag
CD44	Insert CT FLAG tag to CmdT in pCD1-*cmdTAC* from pBAD30-*cmdT*$_{CT\text{-}FLAG}$*A*	ctgctgacgagtaaatgaaaccat ctgttcc

CD45	Insert NT His6 to CmdT in pBAD30-*cmdTA*	tcaggagggattgagctcgtgcaccatcaccatcaccatcaggtagttcatatagggttt
CD46	Insert NT His6 to CmdT in pBAD30-*cmdTA*	aaaccctatatgaactacctgatggtgatggtgatggtgcacgagctcaatccctcctga
CV107	Remove CmdA from pBAD30-*cmdT*$_{NT\text{-}FLAG}$*A*	ttatttatcatcatcatctttataatcaaccttctcacagaaatc
CV108	Remove CmdA from pBAD30-*cmdT*$_{NT\text{-}FLAG}$*A*	/5Phos/aagcttggctgttttggcg
CV109	mutate CmdT with Y41A	ggccctaaacaatggttaactcaaggttacgcgttttggactgatgatccctactgggcccat
CV110	mutate CmdT with Y41A	atgggcccagtagggatcatcagtccaaaacgcgtaaccttgagttaaccattgtttagggcc
CV111	Insert CmdTA into pAJM677	actagagaaagaggggaaatactagatgattgtgcaggtagttcatatag
CV112	Insert CmdTA into pAJM677	ctcttttctggaatttggtaccgagtcaggccgctaaatccaattc
CV113	linearize pAJM677	ctcggtaccaaattccag
CV114	linearize pAJM677	ctagtatttcccctctttc
CV115	CmdTA3xHA fragment	tgagaaggtttaaggagggaaaatgtacccctatgatgtcccggattatgcttacccctatgacgtaccggattacgcgtacccctatgatgtccctgactacgctggcggaggatcgggcggcgggtctctttcctttgaacaaagaatggcct

CV116	Linearize pAJM677-*cmdTA* to insert gBlock	cgtcacactttgctatgcca
CV117	Linearize pAJM677-*cmdTA* to insert gBlock	cgccattggaccaaaacgaa
CV118	pCas9 spacer	aaacatataaacttcaatttttacg
CV119	pCas9 spacer	aaaacgtaaaaattgaagtttatat
CV120	Insert NT HIS into CmdC in pCD1-*cmdTAC*	caccatcaccatcaccataagctaagattaagggataatactgttaatcg
CV121	Insert NT FLAG into CmdC in pCD1-*cmdTAC*	gattataaagatgatgatgataaaaagctaagattaagggataatactgttaatcg
CV122	Insert NT FLAG/HIS into CmdC in pCD1-*cmdTAC*	/5-phos/catcgtcaggccgctaaa
CV123	Amplify vector $CmdT_{CT\text{-}FLAG}AC_{NT\text{-}His6}$ for CmdA HA tag	cgatgcatcaccatcacc
CV124	Amplify vector $CmdT_{CT\text{-}FLAG}AC_{NT\text{-}His6}$ for CmdA HA tag	tttccctccttatttatcatcatc
CV125	Amplify CV115 for 3XHA CmdA tag insertion in $CmdT_{CT\text{-}FLAG}AC_{NT\text{-}His6}$	tgatgatgataaataaggagggaaaatgtacccctatgatgtcc
CV126	Amplify CV115 for 3XHA CmdA tag insertion in $CmdT_{CT\text{-}FLAG}AC_{NT\text{-}His6}$	ttgtgatggtgatggtgatgcatcgtcaggccgctaaatccaattc
CV127	Amplify vector for 3XHA tag insertion into CmdA	ctttcctttgaacaaagaatg
CV128	Amplify vector for 3XHA tag insertion into CmdA	cattttccctccttaaacc

CV129	Model mRNA substrate	ggcaaggagguaaaaaugguu aaa
CV130	Model mRNA reverse complement	aaauugguaaaaauggaggaac gg
CV131	Model DNA substrate	ggcaaggaggtaaaaatggttaa a
CV132	Model DNA reverse complement	tttaaccatttttacctccttgcc
CV133	no-A substrate	ugcugcugcugccgucgucgu cgu
CV134	no-U substrate RNA	agcagcagcagccgacgacgac ga
CV135	no-C substrate RNA	augaugaugaugguaguagua gua
CV136	No-G substrate RNA	aucaucaucauccuacuacuac ua
CV137	specificity_AG ssRNA	tctctctagctctctcagtctctct
CV138	specificity_GA ssRNA	ctctctgactctctcgatctctct
CV139	specificity_AA ssRNA	tctctctaactctctcaatctctct
CV140	specificity_GG ssRNA	tctctctggctctctcggtctctct
CV141	specificity_CC ssRNA	tctctctccctctctccctctctct

SI Table 3: Strains

**Bacterial Strains**		
**Name/Identifier**	**Genotype**	**Source**
ML6	MG1655	
ML3208	MG1655 Δ*clpP*	LeRoux et al., 2020
ML3701	MG1655 pCD1 -*cmdTAC*	Vassallo et al. 2022
ML3717	ECOR22	Ochman, 1984
ML3718	ECOR22 Δ*cmdTAC::kan*	Vassallo et al. 2022
ML3723	MG1655 pBAD30-EV	Vassallo et al. 2022
ML3832	MG1655 pJB37-*darT1*	LeRoux et. al 2022
ML4201	ECOR22 Δ*cmdTAC::kan* pCD1-*cmdTAC*	This study
ML4202	MG1655 pCD1 -$cmdT_{\text{Y41A}}$ *AC*	This study
ML4203	MG1655 pCD1-EV	This study
ML4204	MG1655 pBAD30-*cmdT*	This study
ML4205	MG1655 pBAD30-*cmdA*	This study
ML4206	MG1655 pBAD30-*cmdC*	This study
ML4207	MG1655 pBAD30-*cmdTA*	This study
ML4208	MG1655 pBAD30-$cmdT_{\text{Y41A}}A$	This study
ML4209	MG1655 pBAD30-*cmdTAC*	This study
ML4210	MG1655 pBAD30-$cmdT_{\text{CT -FLAG}}$	This study
ML4211	MG1655 pBAD30-$cmdT_{\text{CT-FLAG}}A$	This study
ML4212	MG1655 pAJM677-$cmdTA_{\text{NT-3xHA}}$	This study
ML4213	MG1655 Δ*clpP* pAJM677-$cmdTA_{\text{NT-3xHA}}$	This study
ML4214	MG1655 Δ*clpP* pCD1-EV pBAD30-EV	This study
ML4215	MG1655 Δ*clpP* pCD1-EV pBAD30-*clpP*	This study
ML4216	MG1655 Δ*clpP* pCD1-*cmdTAC* pBAD30-EV	This study
ML4217	MG1655 Δ*clpP* pCD1-*cmdTAC* pBAD30-*clpP*	This study
ML4218	MG1655 pAJM677-$cmdTA_{\text{NT-3xHA}}$, pIF-EV	This study
ML4219	MG1655 pAJM677-$cmdTA_{\text{NT-3xHA}}$, pIF-*cmdC*	This study
ML4220	MG1655 pCD1-$cmdT_{\text{CT-FLAG}}A_{\text{NT-3xHA}}C_{\text{NT-His6}}$	This study
ML4221	MG1655 pCV38-$cmdT_{\text{CT-FLAG}}A_{\text{NT-3xHA}}C_{\text{NT-His6}}$ pIF1-*EV*	This study
ML4222	MG1655 pCV38-$cmdT_{\text{CT-FLAG}}A_{\text{NT-3xHA}}C_{\text{NT-His6}}$ pIF1-*cmdC*	This study

ML4223	MG1655 pCD1-EV pBAD30-*alt.-3*	This study
ML4224	MG1655 pCD1-*cmdTAC* pBAD30-*alt.-3*	This study
ML4225	MG1655 pAJM677-*cmdTA*$_{\text{NT-3xHA}}$ pIF-*alt.-3*	This study
ML4226	MG1655 pAJM677-*cmdTA*$_{\text{NT-3xHA}}$ pIF-*alt.3*†	This study
ML4227	MG1655 pCD1-*cmdTAC*$_{\text{NT-FLAG}}$	This study
ML4228	MG1655 pCD1-*cmdTAC* pCD16-*gp23* pKVS45-*gp31*	This study
ML4229	MG1655 pCD1-*cmdTAC* pCD16-EV pKVS45-*gp31*	This study
ML4230	MG1655 pCD1-*cmdTAC* pCD16-*gp23* pKVS45-EV	This study
ML4231	MG1655 pCD1-EV pCD16-*gp23* pKVS45-EV	This study
ML4232	MG1655 pBAD30-*cmdT*$_{\text{NT-His6}}$*A*	This study
ML4233	MG1655 Δ*mcrA*Δ*mcrBC* pCas9	This study
ML4234	MG1655 Δ*mcrA*Δ*mcrBC* pCas9-*alt.-3* spacer 2	This study
ML4235	MG1655 Δ*mcrA*Δ*mcrBC,* pCD1-*EV*	This study, parent strain provided by S. Srikant
ML4236	MG1655 Δ*mcrA*Δ*mcrBC,* pCD1-*cmdTAC*	This study, parent strain povided by S. Srikant
ML4315	MG1655 Δ*ompC*::Km^{r}	This study
ML3693	MG1655 pCV1-*PD-T4-1*	Vassallo et al. 2022
**Phage Strains**		
pML31	T4	Guegler and Laub, 2021 (ref[15])
pML55	T4 *alt.-3*†	This study
N/A	T4 Δ*agt* Δ*bgt*	Provided by S. Srikant
pML56	T4 Δ*agt* Δ*bgt* Δ*alt.-3*	This study
pML47	Bas08	(Maffei et al., 2021), DSM #: 112886
pML57	Bas15	(Maffei et al., 2021), DSM #: 112893
pML58	Bas20	(Maffei et al., 2021), DSM #: 112898
pML59	Bas25	(Maffei et al., 2021), DSM #: 112903
pML60	Bas30	(Maffei et al., 2021), DSM #: 112908
pML61	Bas35	(Maffei et al., 2021), DSM #: 112913
pML62	Bas36	(Maffei et al., 2021), DSM #: 112914

pML63	Bas37	(Maffei et al., 2021), DSM #: 112915
pML64	Bas38	(Maffei et al., 2021), DSM #: 112916
pML65	Bas39	(Maffei et al., 2021), DSM #: 112917
pML66	Bas40	(Maffei et al., 2021), DSM #: 112918
pML67	Bas41	(Maffei et al., 2021), DSM #: 112919
pML68	Bas42	(Maffei et al., 2021), DSM #: 112920
pML69	Bas43	(Maffei et al., 2021), DSM #: 112921
pML70	Bas44	(Maffei et al., 2021), DSM #: 112922
pML71	Bas45	(Maffei et al., 2021), DSM #: 112923
pML72	Bas46	(Maffei et al., 2021), DSM #: 112924
pML73	Bas50	(Maffei et al., 2021), DSM #: 112928
pML74	Bas60	(Maffei et al., 2021), DSM #: 112938
pML75	Bas61	(Maffei et al., 2021), DSM #: 112939
pML76	Bas62	(Maffei et al., 2021), DSM #: 112940
pML77	Bas63	(Maffei et al., 2021), DSM #: 112941
pML78	Bas69	(Maffei et al., 2021), DSM #: 112942

SI Table 4: Plasmids

Name	Description	Source
pCD2	pCD1-*cmdTAC*	Vassallo et al., 2022
pCV38	pCD1 -*cmdT* $_{\mathrm{Y41A}}$ *AC*	This study
pCD1	pCD1-EV	Vassallo et al., 2022
pBAD30	pBAD30-EV	Vassallo et al., 2022
pCD8	pBAD30-*cmdT*	This study
pCD9	pBAD30-*cmdA*	This study
pCD10	pBAD30-*cmdC*	Vassallo et al., 2022
pCD4	pBAD30-*cmdTA*	Vassallo et al., 2022
pCV39	pBAD30-*cmdT*$_{\mathrm{Y41A}}$*A*	This study
pCD8	pBAD30-*cmdTAC*	This study
pCD9	pBAD30-*cmdT*$_{\mathrm{CT\text{ -}FLAG}}$	This study
pCD10	pBAD30-*cmdT*$_{\mathrm{CT\text{-}FLAG}}$*A*	This study
pCV40	pAJM677-EV	Meyer et. al 2019
pCV41	pAJM677-*cmdTA*$_{\mathrm{NT\text{-}3xHA}}$	This study
pIF	pIF-EV	Frumkin and Laub, 2023
pCD11	pBAD30-*clpP*	This study
pCD12	pIF-*cmdC*	This study
pCV42	pCD1-*cmdT*$_{\mathrm{CT\text{-}FLAG}}$*A*$_{\mathrm{NT\text{-}3xHA}}$*C*$_{\mathrm{NT\text{-}His6}}$	This study
pCV43	pCD1-*cmdTA*$_{\mathrm{NT\text{-}3xHA}}$*C*	This study
pCD13	pBAD30-*alt.-3*	This study
pCD14	pIF-*alt.-3*	This study
pCD15	pIF-*alt.3*†	This study
pCV44	pCD1-*cmdTAC*$_{\mathrm{NT\text{-}FLAG}}$	This study
pCD16	pCD16-EV	This study
	pKVS45-EV	Frumkin and Laub, 2023
pCD17	pCD16-*gp23*	This study
pCD18	pKVS45-*gp31*	This study
	pJB37-darT1	This study
pCD19	pBAD30-*cmdT*$_{\mathrm{NT\text{-}6xHis}}$*A*	This study
	pCas9	(Jiang et. al, 2013), Addgene no.42876
pCV45	pCas9-alt.-3$_{\mathrm{spacer}}$	This study
pCD20	pCD1-*cmdT*$_{\mathrm{CT\text{-}FLAG}}$*AC*	This study
