# Supplementary material for: Anti-viral defence by an mRNA ADP-ribosyltransferase that blocks translation
Source: Nature. 2024 Oct 23;636(8041):190–7. doi: 10.1038/s41586-024-08102-8 (PMC11618068; doi:10.1038/s41586-024-08102-8)
Supplement: Supplementary file 2 — Reporting Summary [file 41586_2024_8102_MOESM2_ESM.pdf]

Corresponding author(s): Michael Laub

Last updated by author(s): Aug 18, 2024

# Reporting Summary

Nature Portfolio wishes to improve the reproducibility of the work that we publish. This form provides structure for consistency and transparency in reporting. For further information on Nature Portfolio policies, see our Editorial Policies and the Editorial Policy Checklist.

## Statistics

For all statistical analyses, confirm that the following items are present in the figure legend, table legend, main text, or Methods section.

| n/a | Confirmed | |
|---|---|---|
| ☐ | ☒ | The exact sample size (*n*) for each experimental group/condition, given as a discrete number and unit of measurement |
| ☐ | ☒ | A statement on whether measurements were taken from distinct samples or whether the same sample was measured repeatedly |
| ☐ | ☒ | The statistical test(s) used AND whether they are one- or two-sided<br>*Only common tests should be described solely by name; describe more complex techniques in the Methods section.* |
| ☒ | ☐ | A description of all covariates tested |
| ☒ | ☐ | A description of any assumptions or corrections, such as tests of normality and adjustment for multiple comparisons |
| ☐ | ☒ | A full description of the statistical parameters including central tendency (e.g. means) or other basic estimates (e.g. regression coefficient) AND variation (e.g. standard deviation) or associated estimates of uncertainty (e.g. confidence intervals) |
| ☐ | ☒ | For null hypothesis testing, the test statistic (e.g. *F*, *t*, *r*) with confidence intervals, effect sizes, degrees of freedom and *P* value noted<br>*Give P values as exact values whenever suitable.* |
| ☒ | ☐ | For Bayesian analysis, information on the choice of priors and Markov chain Monte Carlo settings |
| ☒ | ☐ | For hierarchical and complex designs, identification of the appropriate level for tests and full reporting of outcomes |
| ☒ | ☐ | Estimates of effect sizes (e.g. Cohen's *d*, Pearson's *r*), indicating how they were calculated |

*Our web collection on statistics for biologists contains articles on many of the points above.*

## Software and code

Policy information about availability of computer code

| | |
|---|---|
| Data collection | Biotek Gen5 v. 3.02 for growth curve data.<br>DefenseFinder v1.3.0 for CmdT logo creation.<br>HMMER3 v3.3.2 for CmdTAC taxonomic distribution. |
| Data analysis | ChimeraX v1.7, bowtie2 v2.3.4.1, cutadapt v1.15, samtools v1.7 genomearray3, pysam v0.16.0.1, pandas v2.0.3, numpy v1.24.4, matplotlib v3.2.2, seaborn v0.10.1, scipy v1.5.0, biopython v1.77, Geneious version 2020.2.4 |

For manuscripts utilizing custom algorithms or software that are central to the research but not yet described in published literature, software must be made available to editors and reviewers. We strongly encourage code deposition in a community repository (e.g. GitHub). See the Nature Portfolio guidelines for submitting code & software for further information.

## Data

Policy information about availability of data

All manuscripts must include a data availability statement. This statement should provide the following information, where applicable:

- Accession codes, unique identifiers, or web links for publicly available datasets
- A description of any restrictions on data availability
- For clinical datasets or third party data, please ensure that the statement adheres to our policy

Summary spectra hits and raw data for IP-MS/MS of CmdC and CmdT pulldowns were deposited at MassIVE and can be accessed at doi:10.25345/C52J68G0H. Raw

data for nucleotide MS and ESI-MS/MS were deposited at MassIVE and can be accessed at doi:10.25345/C51N7XZ2Q. RNA-seq and RIP-seq data are available at GEO under accession number GSE253514.

## Research involving human participants, their data, or biological material

Policy information about studies with human participants or human data. See also policy information about sex, gender (identity/presentation), and sexual orientation and race, ethnicity and racism.

| | |
|---|---|
| Reporting on sex and gender | N/A |
| Reporting on race, ethnicity, or other socially relevant groupings | N/A |
| Population characteristics | N/A |
| Recruitment | N/A |
| Ethics oversight | N/A |

Note that full information on the approval of the study protocol must also be provided in the manuscript.

# Field-specific reporting

Please select the one below that is the best fit for your research. If you are not sure, read the appropriate sections before making your selection.

☒ Life sciences ☐ Behavioural & social sciences ☐ Ecological, evolutionary & environmental sciences

For a reference copy of the document with all sections, see nature.com/documents/nr-reporting-summary-flat.pdf

# Life sciences study design

All studies must disclose on these points even when the disclosure is negative.

| | |
|---|---|
| Sample size | All experiments were performed in at least triplicate with the exception of RNA-seq, RIP-seq, and radiolabel incorporation experiments which were performed in duplicate and IP-MS/MS experiment which were performed once. The majority of experiments were performed in triplicate because of the large effect sizes as a means of identifying reproducibility. RNA sequencing based experiments were performed in duplicate both due to the high cost of these experiments and the large amounts of data produced through these methods. Radiolabel incorporation experiments were performed in duplicate due to the large effect size and inherent qualitative nature of the result. IP-MS/MS experiments were not repeated due to their high time and monetary costs but results were verified through independent means (i.e. activation of CmdTAC by Gp23 as shown in Fig. 2i and Extended Data Fig. 5c. |
| Data exclusions | No data were excluded. |
| Replication | All experimental findings were repeated at least twice. All report results were successfully reported. |
| Randomization | All experiments were performed in isogenic strains so there were no covariates to control for. No subjective choice of experimental and control groups was performed. |
| Blinding | Blinding was not relevant because all data were discrete and/or raw data is reported in the manuscript |

# Reporting for specific materials, systems and methods

We require information from authors about some types of materials, experimental systems and methods used in many studies. Here, indicate whether each material, system or method listed is relevant to your study. If you are not sure if a list item applies to your research, read the appropriate section before selecting a response.

## Materials & experimental systems

| n/a | Involved in the study |
| --- | --- |
| ☐ | ☒ Antibodies |
| ☒ | ☐ Eukaryotic cell lines |
| ☒ | ☐ Palaeontology and archaeology |
| ☒ | ☐ Animals and other organisms |
| ☒ | ☐ Clinical data |
| ☒ | ☐ Dual use research of concern |
| ☒ | ☐ Plants |

## Methods

| n/a | Involved in the study |
| --- | --- |
| ☒ | ☐ ChIP-seq |
| ☒ | ☐ Flow cytometry |
| ☒ | ☐ MRI-based neuroimaging |

# Antibodies

Antibodies used

Poly/Mono-ADP Ribose (E6F6A) Rabbit mAb, Cell Signaling Technologies, Cat #: 83732
6x-His Tag Monoclonal Antibody (HIS.H8), Thermo Fisher, Cat #: MA1-21315
HA-Tag (C29F4) Rabbit mAb, Cell Signaling Technologies, Cat #: 3724S,
DYKDDDDK Tag (D6W5B) Rabbit mAb, Cell Signaling Technologies, Cat #: 14793S
Goat anti-Mouse IgG (H+L) Secondary Antibody, HRP, Thermo Fisher, Cat #: 32430
Goat anti-Rabbit IgG (H+L) Secondary Antibody, HRP, Thermo Fisher, Cat #: 32460

Validation

Antibodies have been validated by manufacturer as listed on website or in listed publications:
Poly/Mono-ADP Ribose: Bullen et al., An ADP-ribosyltransferase toxin kills bacterial cells by modifying structured non-coding RNAs Mol. Cell (2022) DOI:https://doi.org/10.1016/j.molcel.2022.08.015
anti-His: from manufacturer "verified by Relative expression to ensure that the antibody binds to the antigen stated"
anti-HA: validated by "Western blot analysis of extracts from HeLa cells, untransfected or transfected with either HA-FoxO4 or HA-Akt3, using HA-Tag (C29F4) Rabbit mAb.
anti-DYKDDDDK: validated by "Western blot analysis of extracts from 293T cells, mock transfected or transfected with DYKDDDDK-GFP.
anti-Mouse: validated by "Western blot analysis of HA Epitope Tag performed by various amounts of E. coli lysate containing a multi-epitope tagged protein.
anti-Rabbit: validated by "Western blot analysis performed on membrane enriched extracts of K562 and PC-3."

# Plants

Seed stocks

*Report on the source of all seed stocks or other plant material used. If applicable, state the seed stock centre and catalogue number. If plant specimens were collected from the field, describe the collection location, date and sampling procedures.*

Novel plant genotypes

*Describe the methods by which all novel plant genotypes were produced. This includes those generated by transgenic approaches, gene editing, chemical/radiation-based mutagenesis and hybridization. For transgenic lines, describe the transformation method, the number of independent lines analyzed and the generation upon which experiments were performed. For gene-edited lines, describe the editor used, the endogenous sequence targeted for editing, the targeting guide RNA sequence (if applicable) and how the editor was applied.*

Authentication

*Describe any authentication procedures for each seed stock used or novel genotype generated. Describe any experiments used to assess the effect of a mutation and, where applicable, how potential secondary effects (e.g. second site T-DNA insertions, mosiacism, off-target gene editing) were examined.*
